# Overweight Is an Independent Risk Factor for Reduced Lung Volumes in Myotonic Dystrophy Type 1

**DOI:** 10.1371/journal.pone.0152344

**Published:** 2016-03-25

**Authors:** Charlotte G. W. Seijger, Gea Drost, Joram M. Posma, Baziel G. M. van Engelen, Yvonne F. Heijdra

**Affiliations:** 1 Department of Pulmonary diseases, Radboud University Medical Center, Nijmegen, the Netherlands; 2 Department of Neurology, Department of Neurosurgery, University of Groningen, University Medical Center Groningen, Groningen, The Netherlands; 3 Division of Computational and Systems Medicine, Department of Surgery and Cancer, Faculty of Medicine, South Kensington Campus, Imperial College London, London, United Kingdom; 4 Department of Epidemiology and Biostatistics, School of Public Health, Faculty of Medicine, St. Mary’s Campus, Imperial College London, London, United Kingdom; 5 Neuromuscular Center Nijmegen, Institute of Neurology, Radboud University Medical Center, Nijmegen, The Netherlands; Children's Hospital Los Angeles, UNITED STATES

## Abstract

**Background:**

In this large observational study population of 105 myotonic dystrophy type 1 (DM1) patients, we investigate whether bodyweight is a contributor of total lung capacity (TLC) independent of the impaired inspiratory muscle strength.

**Methods:**

Body composition was assessed using the combination of body mass index (BMI) and fat-free mass index. Pulmonary function tests and respiratory muscle strength measurements were performed on the same day. Patients were stratified into normal (BMI < 25 kg/m^2^) and overweight (BMI ≥ 25 kg/m^2^) groups. Multiple linear regression was used to find significant contributors for TLC.

**Results:**

Overweight was present in 59% of patients, and body composition was abnormal in almost all patients. In overweight patients, TLC was significantly (*p* = 2.40×10^−3^) decreased, compared with normal-weight patients, while inspiratory muscle strength was similar in both groups. The decrease in TLC in overweight patients was mainly due to a decrease in expiratory reserve volume (ERV) further illustrated by a highly significant (*p* = 1.33×10^−10^) correlation between BMI and ERV. Multiple linear regression showed that TLC can be predicted using only BMI and the forced inspiratory volume in 1 second, as these were the only significant contributors.

**Conclusions:**

This study shows that, in DM1 patients, overweight further reduces lung volumes, as does impaired inspiratory muscle strength. Additionally, body composition is abnormal in almost all DM1 patients.

## Introduction

Myotonic dystrophy type 1 (DM1) is the most frequent adult-onset muscular dystrophy, with an estimated prevalence of 1 in 8000 individuals [1]. It is a hereditary neuromuscular disorder with progressive muscle weakness and myotonia, caused by an unstable cytosine-thymine-guanine (CTG) repeat expansion in the 3′-untranslated region of the dystrophia myotonica protein kinase (DMPK) gene [2, 3]. Also, several other organs can be involved, resulting in internal, cardiac and respiratory pathology [4].

Aside from effects on daily quality of life due to muscle weakness [4] and fatigue [5], DM1 patients are also at increased risk for physical inactivity and being overweight [6]. In a recent study, overweight, defined as 25 kg/m^2^ ≤ body mass index (BMI) < 30 kg/m^2^, was found in 32.5% of DM1patients, and obesity, defined as BMI ≥ 30 kg/m^2^, was found in 21% of DM1 patients [6]. As DM1 patients typically have reduced muscle mass, overweight will therefore result in increased fat mass [7].

Life expectancy is markedly reduced in DM1 patients; the mean age at death is 54 years [8–10]. The most common cause of death is respiratory failure, approximately in 50% of cases, caused by pneumonia and overall progression of the neuromuscular disorder. Factors increasing the risk of respiratory failure described in DM1 patients include reduced lung volumes, respiratory muscle weakness, swallowing problems, (aspiration) pneumonia and obstructive sleep apnea syndrome [8, 11–13].

Lung volumes of DM1 patients are typically described by a restrictive pattern [13], believed to be caused by impaired respiratory muscle strength; however, in the healthy population, overweight and obesity are also associated with reduced lung volumes [14]. Therefore, the aim of our study is to investigate the additional effects of overweight on lung volumes in DM1 patients, independent of impaired respiratory muscle strength.

## Methods

In this retrospective observational study, body composition and pulmonary function tests were obtained in DM1 patients who visited a chest physician of the Radboud University Medical Center Nijmegen, the Netherlands, in the period between January 2005 and February 2013. Several tests were performed for clinical use by an analist in the pulmonary function laboratory and collected afterwards by the researchers to analyze the data anonymously.

Retrospectively designed studies do not need approval by the ethics committee, as stated by the ethics committee of the Radboud University Medical Center Nijmegen. Patients who take part in investigations for clinical use automatically agree with the use of their data (anonymized by the main researcher) for clinical research; those patients have to disagree actively if they do not wish that their data will be used for clinical research. Thus, as the present investigations were performed for clinical use, there was no need for the investigators to obtain specific informed consent. Based on the information supplied, the main researcher had access to participant information.

Patients were referred for evaluation of pulmonary function by a neurologist or a multidisciplinary team, which consists of a neurologist, rehabilitation physician, chest physician and cardiologist, as well as several paramedics. Patients are referred to a multidisciplinary team only if they experience limitations in everyday life. Patients with diaphragm paralysis due to neurogenic causes were excluded for this study. DM1 severity was measured by the disease-specific muscular impairment rating scale (MIRS) [15]. A score of 1 reflects no muscular impairment, 2 for minimal signs, 3 for distal weakness, 4 for mild to moderate proximal weakness and 5 for severe proximal weakness.

### Body composition

BMI was calculated, and the fat-free mass was measured by bioelectrical impedance analysis (Bodystat, 1500, Douglas, Isle of Man, UK 1997) [16]. The fat-free mass index (FFMI) was calculated using a standardized equation [17]. FFMI is expressed as percentage of cut-off points, where 100% indicates an FFMI of 16 kg/m^2^ for men and 15 kg/m^2^ for women [18]. Patients were stratified by BMI to define overweight and obesity. As criteria for DM1 patients do not exist yet, six different categories of body composition based on criteria used in patients with Chronic Obstructive Pulmonary Disease (COPD) [19] were defined to investigate the existence of an abnormal body composition: cachexia is defined as BMI < 21 kg/m^2^ and FFMI < 100%; semi-starvation as BMI < 21 kg/m^2^ and FFMI ≥ 100%; normal weight with muscle atrophy as 21 kg/m^2^ ≤ BMI < 25 kg/m^2^ and FFMI < 100%; overweight with muscle atrophy as BMI ≥ 25 kg/m^2^ and FFMI < 100%; no impairment as 21 kg/m^2^ ≤ BMI < 25 kg/m^2^ and FFMI ≥ 100%; overweight with normal muscle mass as BMI ≥ 25 kg/m^2^ and FFMI ≥ 100%.

### Respiratory function

All pulmonary function tests were performed according to the standards of the American Thoracic Society/European Respiratory Society [20–23]. Patients underwent spirometry; static lung volumes were measured by the helium dilution technique and diffusion capacity corrected for alveolar volume (DLCO/VA). Static inspiratory (PImax) and expiratory mouth pressures (PEmax) were also measured.

### Statistical analyses

Data analysis was performed using MATLAB (R2012a, The Mathworks, Natick, MA, USA). Pulmonary function test results have normal distributions and are presented as mean values of predicted (%) ± standard deviation (%). MIRS scores were analyzed using non-parametric tests, and differences in body composition and pulmonary function between patients stratified to their MIRS score were tested using analysis of variance (ANOVA) and t-tests. Differences in pulmonary function between normal-body-weight (BMI < 25 kg/m^2^) and overweight (BMI ≥ 25 kg/m^2^) patients were examined using t-tests. Associations between body composition, respiratory muscle strength and lung volumes were examined using Pearson’s correlation. Multiple linear regression with backward stepwise elimination was used to investigate the importance of body composition and respiratory muscle strength for total lung capacity (TLC). Only patients with measurements for all factors were included in the training model set for multiple linear regression. After the significant contributors had been found, a test validation set was defined as the patients left out of the training set that had measurements for the factors that remained in the model (but had missing data for non-significant factors). The model goodness of fit was assessed using the root-mean-square error of the training set, and the goodness of prediction was assessed using the root-mean-square error of prediction. A significance threshold of *p* = 0.05 was used for all tests.

## Results

### Patient characteristics and body composition

**Fig 1:**
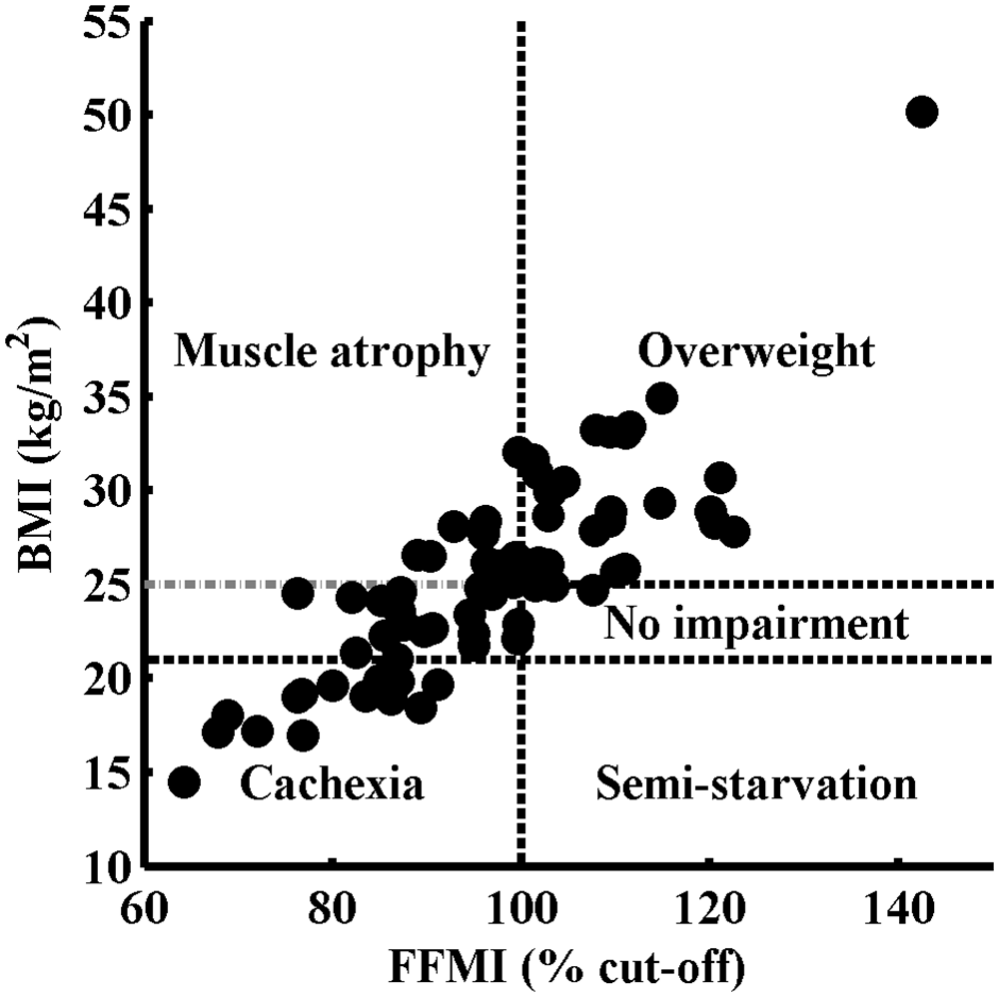
Scatter plot of fat-free mass index (FFMI) and body mass index (BMI) in DM1, n = 71. The x-axis denotes the FFMI, expressed as percentage of gender-specific cut-off points, where 100% indicates FFMI of 16 kg/m^2^ for men and 15 kg/m^2^ for women. The y-axis denotes the BMI, with horizontal lines at 21 and 25 kg/m^2^. The different body compositions are defined as cachexia (BMI < 21 kg/m^2^ and FFMI < 100%), normal weight with muscle atrophy (21 kg/m^2^ ≤ BMI < 25 kg/m^2^ and FFMI < 100%), normal weight with muscle atrophy (BMI ≥ 25 kg/m^2^ and FFMI < 100%), no impairment (21 kg/m^2^ ≤ BMI < 25 kg/m^2^ and FFMI ≥ 100%) and overweight (BMI ≥ 25 kg/m^2^ and FFMI ≥ 100%).

In this study, 106 DM1 patients were analyzed, 59.4% of whom were men. One patient was excluded due to phrenic nerve paralysis. Patient characteristics are shown in Table 1. The median MIRS score was 4, indicating mild to moderate proximal weakness. MIRS 1 is present in 1% of patients, MIRS 2 in 4.8%, MIRS 3 in 22.9%, MIRS 4 in 43.8%, MIRS 5 in 18.1% and for 9.5% of patients scores are missing. In Fig 1, the different categories of body composition are visualized in a scatterplot of FFMI versus BMI for 71 patients, since FFMI was not measured in all patients. No impairment of body composition is present in only 4.2% of patients, cachexia is present in 19.7%, muscle atrophy with normal body weight is present in 26.8%, muscle atrophy with overweight is present in 14.1%, overweight with normal muscle mass is present in 35.2% and none of the patients were classified as semi-starvation. Overweight and obesity, without taking into account FFMI, is present in 41% and 18% of our population, respectively.

**Table 1:** Respiratory function in 105 monotonic dystrophy type 1 (DM1) patients, normal weight (body mass index [BMI] < 25 kg/m^2^) and overweight (BMI ≥ 25 kg/m^2^).

	DM1	BMI < 25 kg/m^2^		BMI ≥ 25 kg/m^2^		
Measurement^a^	Mean in % (SD)^b^	Mean in % (SD)^b^	N = 43^c^	Mean in % (SD)^b^	N = 62^c^	*p*-value^d^
Gender (% men)	60.0%	62.8%	43	58.1%	62	6.27 x 10^−1^
Age (years)	46.2 (12.0)	45.5 (12.4)	43	46.7 (11.8)	62	6.14 x 10^−1^
BMI (kg/m^2^)	26.4 (5.7)	21.7 (2.7)	43	29.6 (5.0)	62	
FFMI (%cut-off)	96.5 (14.3) #	87.0 (10.3)	36	106.3 (10.6)	35	4.96 x 10^−11^ ***
FEV1 (%pred.)	82.9 (18.6) ##	87.3 (16.6) ##	42	79.8 (19.3) ##	61	3.72 x 10^−2^ *
VC (%pred.)	83.9 (18.9) ##	87.7 (17.1)	43	81.2 (19.8)	62	8.58 x 10^−1^
FEV1/VC (%)	101.1 (7.9)	100.9 (8.6)	42	101.4 (7.5)	61	7.55 x 10^−1^
FIV1 (%pred.)	79.6 (21.4) ##	82.4 (20.0) ##	38	78.6 (22.1) ##	60	3.81 x 10^−1^
FRC (%pred.)	72.3 (20.0) ##	86.6 (17.5) ##	39	62.8 (15.4) ##	59	1.52 x 10^−9^ ***
RV (%pred.)	71.1 (16.9) ##	79.8 (19.2) ##	40	65.3 (12.1) ##	59	8.01 x 10^−5^ ***
ERV (%pred.)	73.1 (36.9) ##	98.5 (32.5)	39	60.3 (31.7) ##	59	1.53 x 10^−7^ ***
TLC (%pred.)	79.0 (14.9) ##	84.1 (12.6) ##	41	75.4 (15.4) ##	59	2.40 x 10^−3^ **
DLCO/VA (%pred.)	107.4 (16.2) ##	102.8 (13.1)	38	110.6 (17.4) ##	56	1.49 x 10^−2^ *
PEmax (%pred.)	55.7 (23.5) ##	50.5 (21.3) ##	40	59.6 (24.5) ##	53	5.73 x 10^−2^
PImax (%pred.)	65.0 (23.4) ##	63.5 (23.5) ##	40	66.1 (23.5) ##	51	5.98 x 10^−1^

^a^ Percentage predicted (%pred.) of FFMI (%cut-off): fat-free mass index (kg/m^2^) as percentage of cut-off values (normal values for mean FFMI > 16.0 kg/m^2^ and for women FFMI > 15.0 kg/m^2^); FEV1: forced expiratory volume in 1 second; VC: vital capacity; FIV1: forced inspiratory volume in 1 second; FRC: functional residual capacity; RV: residual volume; ERV: expiratory reserve volume; TLC: total lung capacity; DLCO/VA: diffusion capacity corrected for alveolar volume; PEmax: maximal expiratory mouth pressure; PImax: maximal inspiratory mouth pressure.

^b^ SD: standard deviation;

^#^ *p* < 0.05;

^##^ *p* < 0.001 for a paired t-test between measured and predicted values within each group.

^c^ Number of patients with data available for each measurement.

^d^ *p*-values are given for two-sample t-tests (continuous data) or Chi-squared tests (discrete data), as appropriate, between the groups of BMI.

* *p* < 0.05;

** *p* < 0.01;

*** *p* < 0.001.

### Analyses of respiratory function

Results of pulmonary function tests are presented in Table 1 for all patients and stratified for BMI < 25 kg/m^2^ and BMI ≥ 25 kg/m^2^; potential missing data are indicated by the number of patients for each variable. In both the normal-weight and BMI ≥ 25 kg/m^2^ group, a restrictive pulmonary function pattern is found. Also, respiratory muscle strength, both the inspiratory and expiratory mouth pressures, is decreased significantly compared with their predicted values. The DLCO/VA is normal.

**Fig 2:**
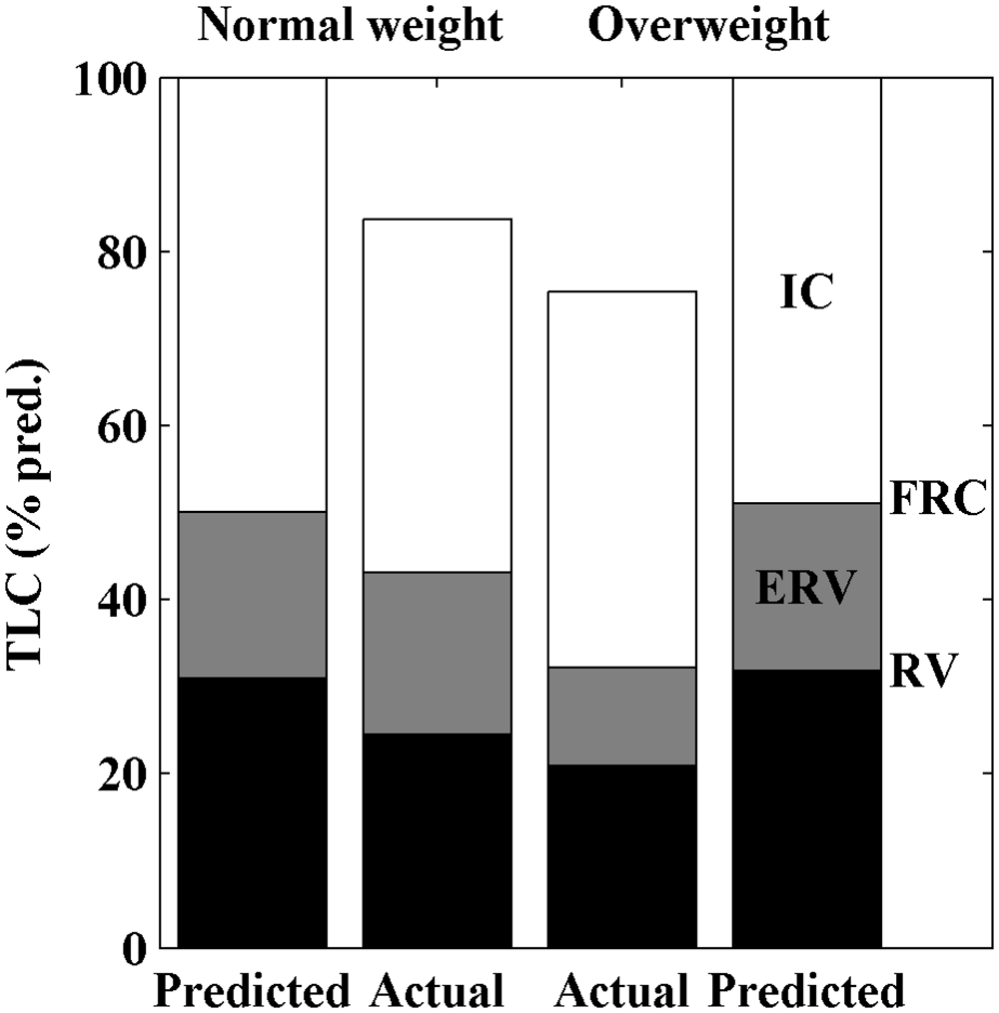
Stacked-bar histogram of total lung capacity (TLC) (% of predicted) in patients with normal weight (body mass index [BMI] < 25 kg/m^2^, n = 43) and overweight (BMI ≥ 25 kg/m^2^, n = 62), compared with their predicted values. The black section of the stacked-bar indicates the residual volume (RV), the gray section the expiratory reserve volume (ERV) and the white section the inspiratory capacity (IC). The RV and ERV combined (black plus grey) is the functional reserve capacity (FRC). A restrictive pattern of pulmonary function is shown for both groups, and the TLC is further decreased in overweight compared with normal-weight patients, mainly due to the decreased ERV.

The TLC, with its different components, is visualized for both groups of BMI in Fig 2. The outer columns show the predicted values. Patients with a BMI ≥ 25 kg/m^2^ have a further reduced TLC compared with patients with BMI < 25 kg/m^2^, with similar values for PImax in both groups. The decrease in TLC in the overweight group is mainly due to the decrease in expiratory reserve volume (ERV). BMI has significant inverse correlations to ERV(r = -0.59 and *p* = 1.33×10^−10^) and functional residual capacity (FRC) (r = -0.61 and *p* = 3.53×10^−11^). PImax is significantly correlated to forced inspiratory volume in 1 second (FIV1) (r = 0.53 and *p* = 1.81×10^−7^) and TLC (r = 0.38 and *p* = 2.04×10^−4^). FIV1 is not correlated to BMI (r = -0.08 and *p* = 4.23×10^−1^). Respiratory function tests between different groups of MIRS score (scores 2 to 5) showed only differences in PEmax, which is significantly higher in MIRS score 3 than in 4 and 5 (*p*-values respectively 2.2×10^−2^ and 1.3×10^−2^). There is no difference in BMI and FFMI between the different MIRS scores.

### Multiple linear regression for TLC

**Fig 3:**
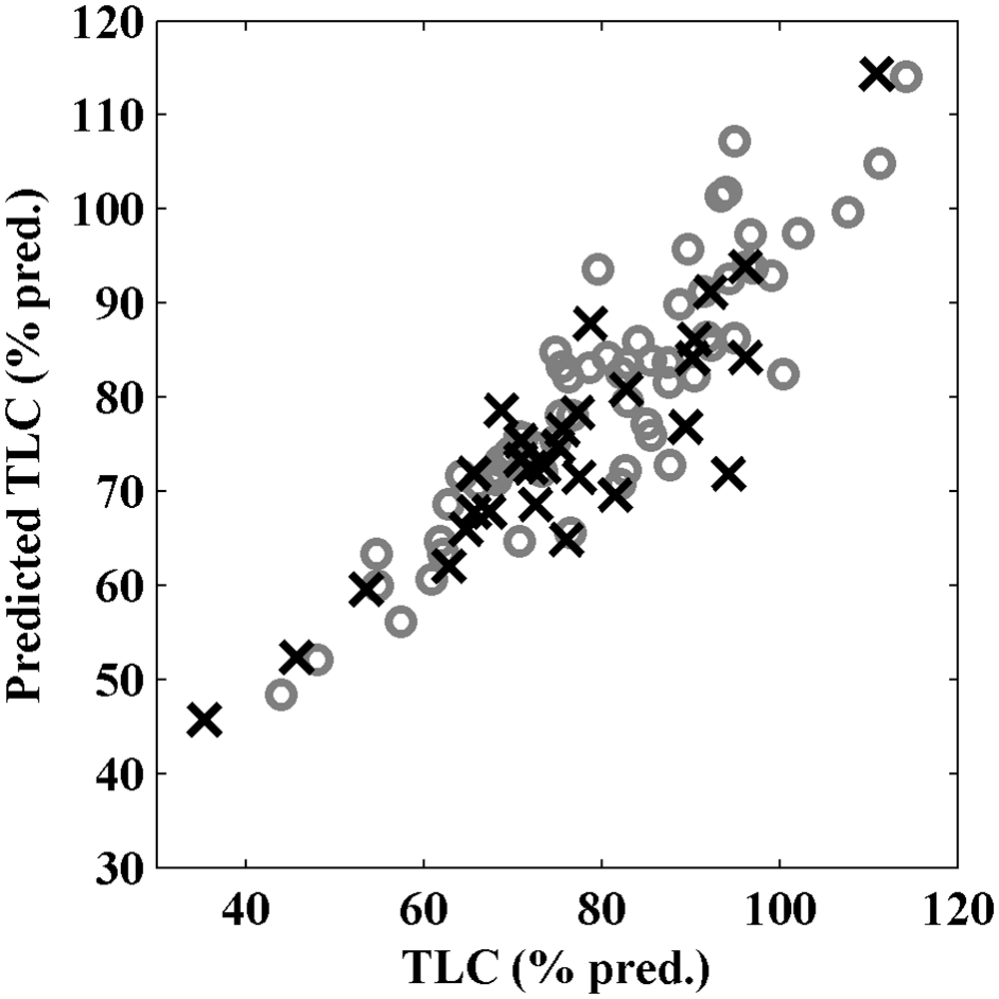
Multiple linear regression model for predicting total lung capacity (TLC) with the significant contributors forced inspiratory volume in 1 second (FIV1) and body mass index (BMI). Patients in the model and validation set are represented by gray circles and black crosses, respectively. The x-axis denotes the TLC expressed as percentage of the predicted value for each individual and the y-axis denotes the calculated predicted TLC (% pred.), based on FIV1 and BMI.

Table 2 shows the correlations of body composition and inspiratory muscle strength parameters with TLC. In order to investigate the joint contribution of these factors to TLC, a multiple linear regression model was calculated. Significant contributors are covariates BMI and FIV1. The model is described by: TLC (%pred.) = 44.54–0.55 × BMI + 0.60 × FIV1 (%pred.). In this model, the *p*-values for BMI and FIV1 are 5.53×10^−4^ and 7.14×10^−23^, respectively. The validation set was defined as the patients left out of the model set who did not have missing data for BMI, TLC and FIV1. The prediction of the validation test set fits well with its expected value (Fig 3). The root-mean-square error of the model set is 6.51% and the root-mean-square error of prediction is 7.39%. This demonstrates that, for an external independent test set, the TLC can be predicted within 7.4% error using only BMI and FIV1 as predictors.

**Table 2:** Pearson’s correlation coefficients for the relation between total lung capacity (TLC) and parameters of body composition and inspiratory muscle strength.

	TLCCorrelation	*p*-value
BMI (kg/m^2^)	-0.27	7.00 x 10^−3^ **
FFMI (%norm)	-0.05	7.13 x 10^−1^
PImax (%pred.)	0.38	2.04 x 10^−4^ ***
FIV1 (%pred.)	0.87	1.97 x 10^−30^ ***

BMI, body mass index; FFMI, fat-free mass index; FIV1: forced inspiratory volume in 1 second; PImax: static inspiratory mouth pressure.

** *p* < 0.01,

*** *p* < 0.001.

## Discussion

The most important findings of this study are that: (1) BMI is a predictor of TLC independent from FIV; (2) TLC is further reduced in the overweight group compared with the normal-weight group due to a reduced ERV, as respiratory muscle strength is equally diminished; and (3) body composition is abnormal in nearly all patients.

In this exceptional large study population of 105 DM1 patients, we found that TLC and FRC are significantly decreased further in overweight patients, compared with normal-weight patients, due to a reduced ERV. This decrease is also found in healthy overweight individuals [14]. Multiple linear regression was used to determine which factors (BMI, FFMI, PImax and/or FIV1) contribute to TLC. Only BMI and FIV1 were found as significant contributors; this means that TLC depends on whole body mass more than on fat-free mass alone. FIV1, which can be seen as a marker of inspiratory strength as it is fully effort dependent, is more accurate in predicting TLC than PImax is. Because we found equally decreased results for PImax and FIV1 in both groups of BMI, the further reduction of TLC in the overweight group is likely to be caused by the additional body weight.

Overweight in general, and therefore also in DM1, results in an increased work of breathing, based on two physiological mechanisms. First, decreased compliance of the thoracic wall [14]. Second, enlargement of tidal volumes can be achieved only by using the inspiratory reserve volume due to reduced ERV level, which will put the respiratory muscles at a disadvantaged position in the Starling curve, where the inspiratory muscles have to generate more tension for equal lung volume enlargement [24]. If increased work of breathing has to be delivered when inspiratory muscle strength is decreased, patients will develop respiratory failure sooner due to muscle fatigue, because the fatigue threshold (PI/PImax) × (T_i_/T_tot_) > 0.15 will be reached earlier [25]. PI is the inspiratory muscle strength needed for normal inspiration and PImax is the maximal inspiratory muscle strength; T_i_ indicates the inspiratory time and the T_tot_ is the total breath-cycle duration. In this study, no difference in PImax between normal-weight and overweight patients was found; thus, the increased work of breathing in overweight patients will result in a higher PI/PImax ratio, and muscle fatigue will develop earlier than in normal weight DM1 patients. To prevent the onset of muscle fatigue and the feelings of dyspnea, patients will decrease tidal volumes, which benefit the work of breathing. However, smaller tidal volumes result in increased dead-space ventilation, early airway closure, atelectasis and a greater likelihood of respiratory infection [26]. Therefore, we argue that overweight DM1 patients are more prone to develop respiratory failure than normal-weight patients.

Losing weight in DM1 patients may possibly result in normalizing lung volumes as is the case in healthy obese individuals [27]. Normalized lung volumes will reduce the work of breathing and might as a result delay the onset of respiratory failure. This hypothesis is valuable for future studies, as respiratory failure is most common cause of death in DM1 [9, 10].

Before starting interventions for weight loss, it will be necessary to investigate the body composition. We found different types of abnormal body composition in nearly all patients. It is difficult to recognize the different types of abnormal body composition using only BMI values. We stratified patients in six categories for body composition based on BMI and FFMI [19]. An abnormal body composition was found in 95.8% of our study population, of whom more than 50% were overweight or obese. Our results regarding BMI alone are in accordance with the results from Gagnon et al. [6]. Physically inactivity, muscle weakness, fatigue as well as, lower socio-economic status, lack of money, limited sports facilities and lack of motivation are known risk factors for overweight in DM1 patients [6]. Inadequate nutritional intake in DM1 patients will also contribute to developing overweight, as fat and carbohydrate intake are often above the daily recommended intake [28]. No prospective observational studies examining body composition changes in DM1 patients have been published to date. We did not find a significant difference between the overweight and normal weight DM1 patients for age.

Body composition is defined by a combination of BMI and FFMI values in 71 patients. Missing data of FFMI are mostly from patients who visited our center in the first years of the study time. A striking outcome of FFMI is that only overweight patients appear to have normal values for FFMI; however, patients with overweight can still have muscle atrophy. A possible explanation can be that skeletal muscles are trained by resistance to the excess weight, and therefore normal values for FFMI are found only in patients with BMI ≥ 25 kg/m^2^. Muscle atrophy could reflect the progression of muscular involvement in DM1 [7]. Pruna et al. [7] investigated body composition with dual-energy X-ray absorptiometry (DEXA) and found comparable results. However, in their population, only one patient had muscle atrophy with overweight, and they found relatively more patients with normal values for both BMI and FFMI. Only three of our patients (4.2%) were considered to have a non-impaired body composition; however, their BMI was very close to 25 kg/m^2^. Based on the MIRS scores, our patients have greater disease severity than the population of Pruna et al.; this might also explain the differences in body composition. These differences in patient characteristics might potentially be explained by our inclusion criteria (see Methods), as severely affected patients are more likely to be referred to a pulmonologist or multidisciplinary team by a neurologist.

Interventions in reducing body weight are ideally based on patients’ individual body composition and must avoid losing muscle mass. A dietitian should ideally be consulted to design a personalized diet, but increasing exercises to lose weight will likely prove to be difficult due to the neuromuscular origin of the disorder. Losing body weight in DM1 patients by increasing exercises requires further investigations. Cup et al. [29] found in different neuromuscular disorders moderate evidence for a combination of muscle-strengthening and aerobic exercises in participation and activities and also in several body functions; however, body weight was not defined as an outcome. In addition, serious adverse effects of exercise were not reported [29]. Overall, the best strategy in reducing body weight will be to prevent the development of overweight.

This large observational study is limited by some missing data and the lack of sniff nasal inspiratory pressure measurements for evaluation of inspiratory muscle strength, which is easier to perform and therefore more preferable than mouth pressures in neuromuscular disorders [30]. PImax maneuvers are more difficult to perform and, especially in patients with neuromuscular disorders, air leaking around the mouthpiece is a possibility. We took precautions to ensure that PImax measurements were included in our data set only if no leaking of air was reported by our laboratory technicians. Results for sniff nasal inspiratory pressure may in general be preferable to PImax measurements. On the other hand, in the evaluation of patients with greater disease severity, sniff nasal inspiratory pressure will possibly underestimate the inspiratory muscle strength, as patients are unable to produce the initial negative trans-nasal pressure to open the nostril valve [30]. Smokers were not excluded in our study, however as smoking may result in an obstructive instead of a restrictive pulmonary function pattern, the results will not be influenced by smoking.

## Conclusion

In DM1 patients, overweight is an independent factor for predicting TLC, and contributes independently of FIV1. Because overweight is related to increased work of breathing and inspiratory muscle strength is reduced in DM1, the fatigue threshold will be reached sooner. Therefore, muscle fatigue and the onset of respiratory failure will develop at an earlier stage in overweight patients, especially during increased ventilator demand. Moreover, over half of DM1 patients are overweight, and nearly all patients have an abnormal body composition. To develop interventional strategies for weight loss, it will be important to categorize the individual type of body composition. Hence, preventing the development of overweight in DM1 patients may result in delaying respiratory failure and mortality in DM1.

## Supporting Information

S1 File Data of body composition and pulmonary function in DM1 patients. (XLSX)

## References

[1] Harper PS. Myotonic Dystrophy. 3rd ed. London, UK: W.B. Saunders; 2001.

[2] Brook JD, McCurrach ME, Harley HG, Buckler AJ, Church D, Aburatani H, et al Molecular basis of myotonic dystrophy: expansion of a trinucleotide (CTG) repeat at the 3' end of a transcript encoding a protein kinase family member. Cell. 1992;69(2):385 1568252 10.1016/0092-8674(92)90418-c

[3] Mahadevan M, Tsilfidis C, Sabourin L, Shutler G, Amemiya C, Jansen G, et al Myotonic dystrophy mutation: an unstable CTG repeat in the 3' untranslated region of the gene. Science. 1992;255(5049):1253–5. 1546325 10.1126/science.1546325

[4] Turner C, Hilton-Jones D. The myotonic dystrophies: diagnosis and management. J Neurol Neurosurg Psychiatry. 2010;81(4):358–67. 10.1136/jnnp.2008.158261 20176601

[5] Kalkman JS, Schillings ML, van der Werf SP, Padberg GW, Zwarts MJ, van Engelen BG, et al Experienced fatigue in facioscapulohumeral dystrophy, myotonic dystrophy, and HMSN-I. J Neurol Neurosurg Psychiatry. 2005;76(10):1406–9. 16170086 10.1136/jnnp.2004.050005 PMC1739364

[6] Gagnon C, Chouinard MC, Laberge L, Brisson D, Gaudet D, Lavoie M, et al Prevalence of lifestyle risk factors in myotonic dystrophy type 1. Can J Neurol Sci. 2013;40(1):42–7. 23250126 10.1017/s0317167100012932

[7] Pruna L, Chatelin J, Pascal-Vigneron V, Kaminsky P. Regional body composition and functional impairment in patients with myotonic dystrophy. Muscle Nerve. 2011;44(4):503–8. 10.1002/mus.22099 21826679

[8] de Die-Smulders CE, Howeler CJ, Thijs C, Mirandolle JF, Anten HB, Smeets HJ, et al Age and causes of death in adult-onset myotonic dystrophy. Brain. 1998;121 (Pt 8):1557–63. 9712016 10.1093/brain/121.8.1557

[9] Groh WJ, Groh MR, Shen C, Monckton DG, Bodkin CL, Pascuzzi RM. Survival and CTG repeat expansion in adults with myotonic dystrophy type 1. Muscle Nerve. 2011;43(5):648–51. 10.1002/mus.21934 21484823

[10] Mathieu J, Allard P, Potvin L, Prevost C, Begin P. A 10-year study of mortality in a cohort of patients with myotonic dystrophy. Neurology. 1999;52(8):1658–62. 10331695 10.1212/wnl.52.8.1658

[11] Kiyan E, Okumus G, Cuhadaroglu C, Deymeer F. Sleep apnea in adult myotonic dystrophy patients who have no excessive daytime sleepiness. Sleep Breath. 2010;14(1):19–24. 10.1007/s11325-009-0270-6 19484280

[12] Begin P, Mathieu J, Almirall J, Grassino A. Relationship between chronic hypercapnia and inspiratory-muscle weakness in myotonic dystrophy. Am J Respir Crit Care Med. 1997;156(1):133–9. 9230737 10.1164/ajrccm.156.1.9509041

[13] Bogaard JM, van der Meche FG, Hendriks I, Ververs C. Pulmonary function and resting breathing pattern in myotonic dystrophy. Lung. 1992;170(3):143–53. 1614221 10.1007/BF00174317

[14] Littleton SW. Impact of obesity on respiratory function. Respirology. 2012;17(1):43–9. 10.1111/j.1440-1843.2011.02096.x 22040049

[15] Mathieu J, Boivin H, Meunier D, Gaudreault M, Begin P. Assessment of a disease-specific muscular impairment rating scale in myotonic dystrophy. Neurology. 2001;56(3):336–40. 11171898 10.1212/wnl.56.3.336

[16] Lukaski HC, Johnson PE, Bolonchuk WW, Lykken GI. Assessment of fat-free mass using bioelectrical impedance measurements of the human body. Am J Clin Nutr. 1985;41(4):810–7. 3984933 10.1093/ajcn/41.4.810

[17] Kyle UG, Genton L, Karsegard L, Slosman DO, Pichard C. Single prediction equation for bioelectrical impedance analysis in adults aged 20–94 years. Nutrition. 2001;17(3):248–53. 11312069 10.1016/s0899-9007(00)00553-0

[18] Baarends EM, Schols AM, Mostert R, Wouters EF. Peak exercise response in relation to tissue depletion in patients with chronic obstructive pulmonary disease. Eur Respir J. 1997;10(12):2807–13. 9493665 10.1183/09031936.97.10122807

[19] Schols AM, Broekhuizen R, Weling-Scheepers CA, Wouters EF. Body composition and mortality in chronic obstructive pulmonary disease. Am J Clin Nutr. 2005;82(1):53–9. 16002800 10.1093/ajcn.82.1.53

[20] Wanger J, Clausen JL, Coates A, Pedersen OF, Brusasco V, Burgos F, et al Standardisation of the measurement of lung volumes. Eur Respir J. 2005;26(3):511–22. 16135736 10.1183/09031936.05.00035005

[21] Macintyre N, Crapo RO, Viegi G, Johnson DC, van der Grinten CP, Brusasco V, et al Standardisation of the single-breath determination of carbon monoxide uptake in the lung. Eur Respir J. 2005;26(4):720–35. 16204605 10.1183/09031936.05.00034905

[22] Miller MR, Hankinson J, Brusasco V, Burgos F, Casaburi R, Coates A, et al Standardisation of spirometry. Eur Respir J. 2005;26(2):319–38. 16055882 10.1183/09031936.05.00034805

[23] American Thoracic Society/European Respiratory S. ATS/ERS Statement on respiratory muscle testing. Am J Respir Crit Care Med. 2002;166(4):518–624. 12186831 10.1164/rccm.166.4.518

[24] Loring SH, Garcia-Jacques M, Malhotra A. Pulmonary characteristics in COPD and mechanisms of increased work of breathing. J Appl Physiol. 2009;107(1):309–14. 10.1152/japplphysiol.00008.2009 19359620 PMC2711781

[25] Bellemare F, Grassino A. Effect of pressure and timing of contraction on human diaphragm fatigue. J Appl Physiol Respir Environ Exerc Physiol. 1982;53(5):1190–5. 7174413 10.1152/jappl.1982.53.5.1190

[26] Schmidt-Nowara WW, Altman AR. Atelectasis and neuromuscular respiratory failure. Chest. 1984;85(6):792–5. 6723392 10.1378/chest.85.6.792

[27] Refsum HE, Holter PH, Lovig T, Haffner JF, Stadaas JO. Pulmonary function and energy expenditure after marked weight loss in obese women: observations before and one year after gastric banding. Int J Obes. 1990;14(2):175–83. 2111293

[28] Motlagh B, MacDonald JR, Tarnopolsky MA. Nutritional inadequacy in adults with muscular dystrophy. Muscle Nerve. 2005;31(6):713–8. 15786416 10.1002/mus.20317

[29] Cup EH, Pieterse AJ, Ten Broek-Pastoor JM, Munneke M, van Engelen BG, Hendricks HT, et al Exercise therapy and other types of physical therapy for patients with neuromuscular diseases: a systematic review. Arch Phys Med Rehabil. 2007;88(11):1452–64. 17964887 10.1016/j.apmr.2007.07.024

[30] Terzi N, Orlikowski D, Fermanian C, Lejaille M, Falaize L, Louis A, et al Measuring inspiratory muscle strength in neuromuscular disease: one test or two? Eur Respir J. 2008;31(1):93–8. 17898014 10.1183/09031936.00094707

